# Hyperspectral Imaging for Lateral Tumour Demarcation of High-risk Basal Cell Carcinomas during Mohs Micrographic Surgery

**DOI:** 10.2340/actadv.v105.43614

**Published:** 2025-12-02

**Authors:** Hannah CEDER, Mari SALMIVUORI, Ilkka PÖLÖNEN, John PAOLI, Noora NEITTAANMÄKI

**Affiliations:** 1 Department of Dermatology and Venereology, Institute of Clinical Sciences, Sahlgrenska Academy, University of Gothenburg, Gothenburg; 2 Region Västra Götaland, Sahlgrenska University Hospital, Department of Dermatology and Venereology, Gothenburg; 3 Department of Dermatology, Allergology and Venereology, Helsinki University Hospital and University of Helsinki, Helsinki; 4 Faculty of Information Technology, University of Jyväskylä, Jyväskylä, Finland; 5 Department of Laboratory Medicine, Institute of Biomedicine, Sahlgrenska Academy, University of Gothenburg, Gothenburg; 6 Department of Pathology, Region Västra Götaland, Sahlgrenska University Hospital, Gothenburg, Sweden

**Keywords:** high-risk basal cell carcinoma, Mohs micrographic surgery, tumour demarcation, hyperspectral imaging

## Abstract

Hyperspectral imaging is a non-invasive imaging modality showing potential in delineating tumour margins preoperatively. This pilot study evaluated the feasibility of using hyperspectral imaging to demarcate lateral margins of high-risk facial basal cell carcinomas (BCC) prior to Mohs micrographic surgery. Thirty patients with high-risk BCCs were recruited from the Department of Dermatology, Sahlgrenska University Hospital, Sweden. Lesions were initially demarcated using dermoscopy, followed by hyperspectral imaging scans. During the first stage, a superficial vertical incision was performed along the demarcation line before adding a 3-mm clinical margin for the bowl-shaped excision of the tumour. Hyperspectral imaging-based tumour margins were compared with histopathologically verified borders, serving as ground truth. The data analysis used supervised learning; 2 complementary validation strategies were employed: a half-split approach where the left half of each annotated image was used for training and the right half for testing, and a leave-one-out cross-validation at the image level. A pixel-wise classification approach was used, treating each pixel as an independent sample. Hyperspectral imaging achieved a pixel-wise classification accuracy of 0.76, sensitivity of 0.75, specificity of 0.78, and an area under the receiver operating characteristic curve of 0.84. Hyperspectral imaging demonstrated potential for tumour demarcation, providing a basis for future research.

## SIGNIFICANCE

Hyperspectral imaging combines digital imaging and spectroscopy for non-invasive, *in vivo* diagnosis of skin cancer. Its advantages include large field of view and rapid imaging process. This pilot study assessed the feasibility of a convolutional neural network trained on hyperspectral images for detecting the lateral borders of high-risk basal cell carcinomas prior to Mohs micrographic surgery. The results indicate that hyperspectral imaging provides relatively high sensitivity and specificity for this task. These results will serve as a basis for future development as well as further validation with larger datasets.

Basal cell carcinoma (BCC) is the most common type of skin cancer and a major public health problem in fair-skinned populations. Although it rarely metastasizes, BCC can cause morbidity due to aggressive and destructive local growth (1). Histopathologically, BCCs can be classified by their morphological growth pattern into low-risk (nodular or superficial) or high-risk subtypes (aggressive growth patterns).

The aggressive growth patterns are infiltrative, micronodular, morpheaform, i.e., sclerotic, basosquamous, i.e., metatypical and perineural or perivascular growth (1–3). Subclinical extension in aggressive BCCs can consist of few cells/thick cords, which increases the risk of incomplete excision in traditional surgery (2, 3). Additionally to the histopathological subtype, there are other clinical factors, which increases the risk of recurrence of BCC. These risk factors are facial location, large diameter, ill-defined borders, incomplete primary excision, and recurrence of the tumour (4, 5). If available, Mohs micrographic surgery (MMS) is the choice of the treatment for BCCs at high-risk of recurrence. MMS provides 100% intraoperative margin control with minimal defect size, and thus is particularly valuable in the facial region, where maintaining anatomical structures and function is essential. With complete surgical removal of the tumour re-excision can be avoided and risk of recurrence reduced (4, 6).

To identify high-risk BCCs, dermoscopy plays an important role (7, 8). A systematic review and meta-analysis demonstrated a pooled sensitivity of 91.2% and specificity of 95% for dermoscopy in diagnosis of BCC (9). Subtyping BCCs with dermoscopy can, however, be more challenging, especially regarding the ability to predict high-risk histopathological subtypes (8). Importantly, delineating tumour borders in high-risk BCCs with dermoscopy prior to MMS has also proved to be difficult (10).

Other non-invasive imaging techniques for BCC demarcation prior to MMS include optical coherence tomography (OCT), reflectance confocal microscopy (RCM), and line-field confocal optical coherence tomography (LC-OCT) (10–14). Previous studies have shown that the preoperative *in vivo* application of RCM and OCT can decrease the number of excision stages required during MMS for BCC (10, 15). LC-OCT has been demonstrated to significantly reduce the number of MMS stages to achieve tumour clearance when used for preoperative evaluation of high-risk BCC margins (16).

Hyperspectral imaging (HI) is a non-invasive technology that allows spectral data to be obtained from an image. A regular camera captures images in 3 broad wavebands (red, green, and blue) while HI uses dozens of continuous narrow wavebands including subtle wavelength differences and certain wavelengths which are outside the range of human vision (460–830 nm). A hyperspectral image is a stack of hundreds of overlapping images taken at different narrow wavebands of light. The resulting hyperspectral cube contains 2 spatial dimensions and the spectral data for every pixel provides a third dimension. This results in a unique spectral graph for different biological tissues (17). The spectra can be presented as abundance maps showing the localization and demarcation of the tumour (17–21). The advantages include large field of view (up to 12 cm^2^) and rapid imaging process. The lateral resolution is 20 μm, which enables the detection of cellular aggregates.

HI has shown potential in the preoperative delineation of lentigo maligna (19) and the detection of field-cancerized skin (20). To date, only 1 study has been performed using HI to delineate the lateral margins of BCCs preoperatively (22). In this pilot study, we aimed to test the feasibility of using supervised learning based on hyperspectral images to detect the lateral margins of high-risk BCCs compared with the histopathologically verified tumour borders observed during MMS.

## MATERIALS AND METHODS

### Recruitment

The study was approved by the Swedish Ethical Review Authority (approval number 2021-04753). All patients were recruited prospectively between January 2022 and January 2023 from the Department of Dermatology, Sahlgrenska University Hospital in Gothenburg, Sweden. Patients with primary facial high-risk BCCs planned for treatment with MMS were informed orally and in writing, and they provided written consent ahead of the surgery. Exclusion criteria were patients who could not give informed consent as well as patients with lesions located on very irregular surfaces (e.g., ear or eyelids). As this was a pilot study, a sample size was not calculated, but we aimed to include approximately 30 tumours.

### Image acquisition and sampling for histopathology

All lesions were first evaluated clinically and with a Dermlite DL200 Hybrid dermatoscope (3Gen, Santa Ana, CA, USA) and later photographed with an iPhone 8 smartphone camera (Apple, Cupertino, CA, USA). Tumour borders detected with dermoscopy were marked on the skin using a felt-tip pen. The dermatoscopic evaluation was done by HC who is a Mohs surgeon, and a specialist in dermatology with more than 10 years of experience. The HI system Cutica prototype (Revenio Group Oyj, Vantaa, Finland) was used to obtain hyperspectral images of the lesion and surrounding healthy skin, prior to and after the lesion borders detected with dermoscopy were marked on the skin.

**Fig. 1:**
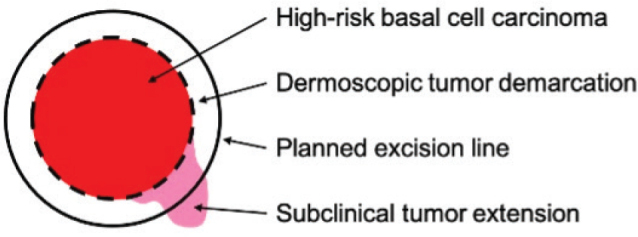
**Verification of subclinical tumour extension.** Clinically and dermoscopically detected tumour area (red), dermoscopic tumour demarcation, and superficial incision line in the specimen (dotted line) added prior to excision according to the principles of Mohs micrographic surgery along the planned excision line (solid line) and hypothetical subclinical tumour extension detected histopathologically (pink).

The BCCs were then excised following the standard MMS protocol. In addition, a superficial incision line was added to the specimen following the clinically and dermoscopically detected tumour demarcation prior to excision. The incision line was used to facilitate comparison between dermoscopically visible tumour borders and histopathologically verified tumour borders. With help from the incision line, the subclinical extension could be verified (**Fig. 1**). The histopathologically verified tumour borders of the MMS specimens represented the true classification (true label) of the lesions when later comparing them with the borders detected in the hyperspectral images.

### The hyperspectral imaging system

**Fig. 2:**
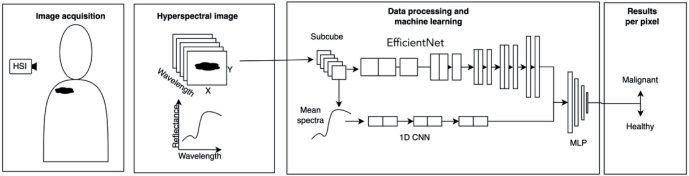
**Data acquisition, formatting, and processing of HSI data.** Each image is segmented into overlapping subcubes centred on each spatial pixel. The subcube is input into a deep neural network model with 2 branches: 1 branch analyses spatial features, while the other examines spectral features. The network’s output is a binary classification indicating the malignancy of the central pixel.

The HI system consists of a Fabry-Pérot interferometer (FPI) based hyperspectral imager and diffuse illumination system (23). The hyperspectral imager captures 120 wavebands rapidly in seconds using the diffuse reflectance of visible and near-infrared light (wavebands 460–830 nm) within a large field of view of 12 cm^2^ (spatial resolution 6,400 pixels/cm) in 5–10 s. In the wavelength range used, the imaging depth varies between 0.5 and 5 mm as a function of wavelength (24). The full width of each waveband’s half maximum varies from 5 to 15 nm. The camera used is capable of taking images at a resolution of 1,920×1,200 pixels. This corresponds to approximately 15 μm/pixel spatial resolution. Thus, aggregates of cells are visible in hyperspectral images. The camera employs an adjustable filter to select the wavelength band to be imaged. After capturing 1 spectral band, a new wavelength range is selected and imaged. Ultimately, these separate images are combined into a 3-dimensional image cube with both spatial and spectral dimensions. Thus, each spatial pixel in the image contains the full spectrum of the targeted skin region (**Fig. 2**). In addition to the spectral data cube, the HI system provided RGB images of the imaged area. A more detailed description is available elsewhere (18, 19, 25).

### Data processing and statistical analysis

**Fig. 3:**
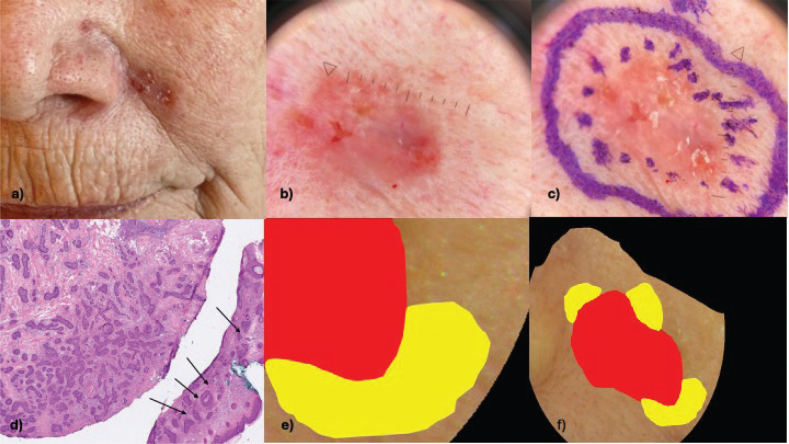
**Examples of the annotation process used for supervised training of the hyperspectral imaging data.** (A) Clinical image of a high-risk infiltrative basal cell carcinoma in the nasolabial fold. (B) Dermoscopic image of the lesion. (C) Dermoscopic image including the tumour demarcation based on the dermoscopic evaluation (dotted line) as well as the planned excision line adding 2–3 mm resection margin (solid line). (D) Histopathological image showing the subclinical extension beyond the dermoscopically detected lateral tumour border demarcation (black arrows). (E) RGB image captured by the hyperspectral camera with annotations for the tumour area seen with dermoscopy (red) and the subclinical extension based on histopathology (yellow). (F) Annotation on an entire lesion with subclinical extension based on histopathology (yellow) in multiple areas not detected with dermoscopy (red).

For data analysis, supervised learning was employed, using annotated images as the ground truth. The annotations were based on the histopathologically verified lesion borders annotated on left halves of the RGB images captured by the HI system (**Fig. 3**). The right halves were kept unannotated for testing.

The study employed a 2-branch convolutional neural network (CNN), similar to those previously described in the literature (26). One branch, based on EfficientNet (a 2D CNN pretrained on ImageNet; https://www.image-net.org/) (27) was utilized to extract spatial features. The other branch, a 1D CNN, focused on extracting features along the spectral dimension. After feature extraction, the outputs of the 2 branches were concatenated into a single feature vector, which was then fed into a neural network responsible for classification. The network input consisted of a 25×25 pixel region centred on each pixel of interest. From this region, 3 spectral bands (at 487, 604, and 795 nm) were selected for the spatial feature extraction branch. These bands were processed by the 2D CNN branch. The 1D CNN branch, in turn, received the mean spectrum from the selected lesion half normalized by subtracting its mean and dividing by its standard deviation.

Due to the limited number of lesions, 2 complementary validation strategies were employed. First, a leave-one-out cross-validation was conducted, where each lesion image was excluded in turn and the model was trained on all remaining images. This provided a conservative baseline estimate of model performance when generalizing to completely unseen lesions. Second, to approximate the scenario of having a larger dataset covering the full variability of lesions, each image was vertically split at its centre (21). The left half of each lesion image was used for training and validation, while the right half was reserved for testing. This ensured that no pixel-level information was shared between training and test sets. Data augmentation, including horizontal and vertical flips and random rotations, was applied during training. The model was implemented using the PyTorch library (https://pytorch.org/). Prior to final training, the model’s hyperparameters were optimized using the Optuna library (https://optuna.org/). The training utilized binary cross-entropy with logits as the loss function and the Adam optimizer. Early stopping criteria were employed to prevent overfitting. Training was conducted on an NVIDIA RTX A4000 GPU (https://www.nvidia.com/en-gb/products/workstations/rtx-a4000/).

### Statistical analysis

Model performance was assessed using the area under the receiver operating characteristic curve (AUC), which quantifies overall discrimination ability, as well as sensitivity and specificity to measure correct identification of positive and negative cases, respectively. Classification outcomes were also visualized by color-coding pixels in each lesion: true negatives in green, true positives in blue, false positives in red, and false negatives in yellow.

## RESULTS

### Lesion characteristics

A total of 36 lesions fulfilling the inclusion criteria were imaged. One lesion was excluded when it was discovered that it was a recurrent BCC and 5 images were excluded due to poor image quality or missing images. Hence, a total of 30 lesions from 30 patients with a median age of 69 years (range 38–86 years) were included in the data analysis. Patient demographics and lesion characteristics are presented in Table I. The median tumour diameter was 13 mm (range 5–21 mm). All the lesions fitted the field of view.

**Table I:** Patient demographics, tumour characteristics. and outcomes following Mohs micrographic surgery

No.	Age, years	Sex	Lesion localization	Lesion size, mm	Subclinical lateral tumour extension
1	55	F	Forehead	21	Yes
2	72	F	Cheek	20	Yes
3	86	M	Nose	10	No
4	81	F	Nose	7	No
5	61	F	Upper lip	5	No
6	47	M	Forehead	10	Yes
7	79	M	Forehead	15	No
8	66	M	Nose	13	No
9	69	F	Cheek	15	Yes
10	74	F	Cheek	15	Yes
11	71	F	Cheek	21	Yes
14	72	F	Nose	20	Yes
15	75	M	Cheek	15	Yes
16	68	M	Eyebrow	17	No
17	83	M	Upper lip	14	No
18	69	F	Temporal	18	No
19	56	F	Nose	8	Yes
20	65	F	Nose	11	Yes
23	82	M	Medial cantus	10	Yes
24	79	F	Cheek	8	No
25	41	F	Eyebrow	12	Yes
26	86	F	Lower lip	10	No
27	76	F	Upper lip	15	Yes
28	38	M	Nose	15	Yes
30	78	F	Eyebrow	10	Yes
31	72	F	Cheek	15	Yes
32	53	F	Temporal	10	No
33	68	M	Cheek	16	Yes
34	82	M	Nose	12	Yes
36	77	F	Upper lip	15	Yes

### Subclinical lateral tumour extension

**Fig. 4:**
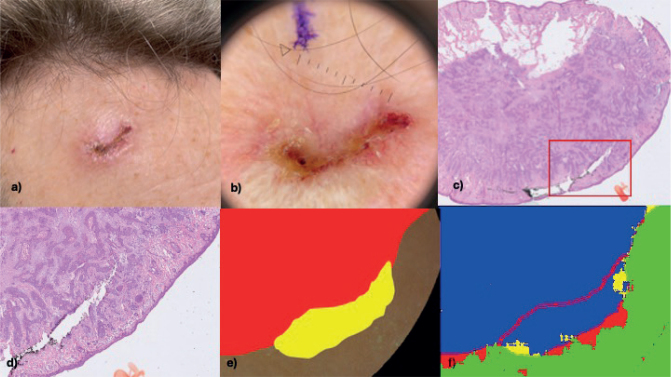
**Hyperspectral analysis results compared with histopathology serving as ground truth.** (A) Clinical image of a patient with an infiltrative basal cell carcinoma on the forehead. (B) Dermoscopic image of the tumour. (C) Histopathologically verified tumour detected during Mohs micrographic surgery with subclinical lateral tumour extension outside the incision line corresponding to the dermoscopic tumour border demarcation (red rectangle). (D) Higher magnification of the area with subclinical lateral tumour extension. (E) Annotation based on histopathology showing subclinical lateral tumour extension (yellow). (F) Hyperspectral imaging analysis showing true negative pixels (green), true positive pixels (blue), false positive pixels (red), and false negative pixels (yellow).

In total, 19/30 lesions showed subclinical lateral tumour extension outside the dermoscopically detected tumour border (**Fig. 4**). In all 19 cases, the lateral margin was involved, and in 1 case the deep margin was involved as well.

### Hyperspectral analysis

Classification outcomes were visualized by colour-coding pixels in each of the lesion halves: true negatives in green, true positives in blue, false positives in red, and false negatives in yellow (see Fig. 4).

**Fig. 5:**
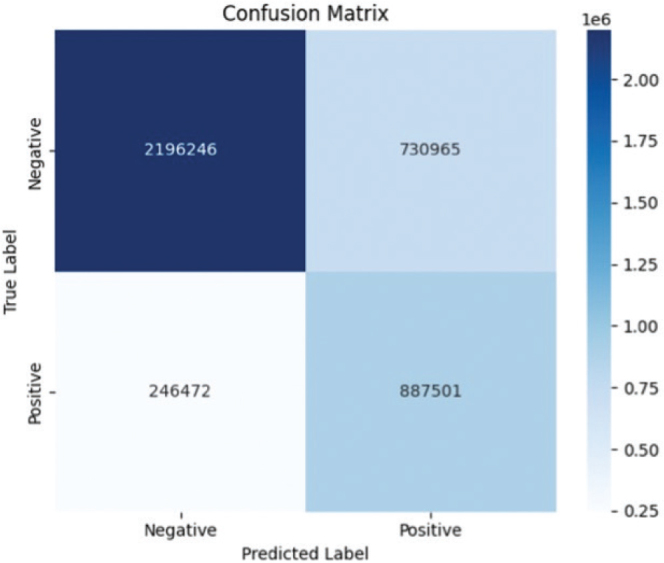
**Pixel-wise classification.** The number of pixels in each class is shown in the confusion matrix.

**Fig. 6:**
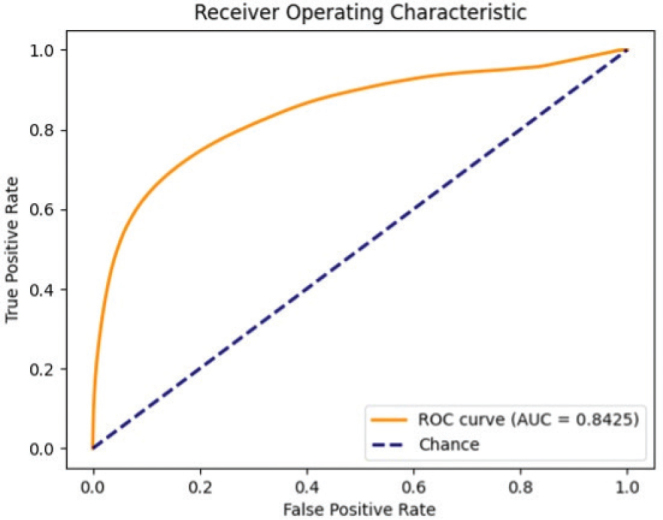
**Receiver operating characteristic (ROC) curve and area under the ROC curve (AUC) obtained when comparing the tumour detection with hyperspectral imaging with histopathology**.

The overall accuracy of the pixel-wise classification in the vertical splitting approach was 0.76 (± 0.0004), with a sensitivity of 0.75 (± 0.0005) and a specificity of 0.78 (± 0.0008) (**Fig. 5**). In total, 4,061,184 pixels were analysed, of which 1,133,973 were positive for tumour. The ROC analysis yielded an AUC of 0.84 (**Fig. 6**).

In addition, leave-one-out cross-validation was performed, where each lesion image was excluded once as the test case. The average results across all folds were accuracy 0.70 (± 0.15), sensitivity 0.52 (± 0.31), specificity 0.80 (± 0.21), and AUC 0.66 (± 0.11). These results represent a conservative estimate of model performance when applied to entirely unseen lesions, whereas the vertical splitting experiment reflects the performance of a model trained on a more diverse dataset covering the full variability of the lesions.

## DISCUSSION

In this pilot study, we studied the feasibility of using *in vivo* HI technology to preoperatively delineate the lateral margins of facial high-risk BCCs. The results of the histopathologically verified tumour extensions observed with MMS were compared with the HI images using supervised learning of a CNN. The CNN analysis used a pixel-wise classification reaching relatively high sensitivity and specificity in detecting subclinical lateral tumour extensions.

The advantage of HI is that it combines digital imaging with spectroscopy. It allows for spectral data extraction from map-like images and offers a large imaging view of 12 cm^2^. Furthermore, the imaging process is rapid, taking only seconds to capture data, and gives automated analysis performing visual data (19, 22). However, the CNN developed for this study is currently not integrated into the HI device. The HI system Cutica prototype (Revenio Group Oyj, Vantaa, Finland) used in the study is currently not in commercial use, which limits accessibility.

Several non-invasive imaging techniques are available for assessing the lateral margins of BCCs, including: dermoscopy, RCM, OCT, and LC-OCT. Of these, dermoscopy is the most used method in clinical practice for detecting lateral BCC margins. However, its effectiveness in reducing the number of excision stages during MMS is disputed and evidence supporting its accuracy is limited (10, 11). Yeom et al. summarize that dermoscopic evaluation is useful for reducing the final number of MMS stages and defect size during MMS, especially in patients with BCC (28). The diagnostic accuracy of dermoscopy is, however, user-dependent (9).

In contrast, preoperative use of RCM and OCT has shown promise in reducing the number of excision stages required during MMS for BCCs (10). These advanced imaging techniques, as well as LC-OCT, may offer more precise margin delineation compared with dermoscopy, potentially improving surgical outcomes and efficiency. In 1 study, 8 out of 10 BCCs were completely excised in a single stage when margin delineation was done by OCT. Macroscopic margins were enlarged after OCT scanning in 4 patients, saving further stages of MMS (15). Paradisi et al. studied LC-OCT and showed a significant difference in mean number of MMS stages when using LC-OCT as well as significantly reducing the patients’ risk of undergoing > 1 MMS stage by preoperative mapping of BCC tumour margins (16). On the other hand, a study by Adan et al. concluded a sensitivity of 63.0% and a specificity of 53% for the assessment of BCC resection margins prior to MMS with OCT (29). For comparison, a study by Venturini et al. found that RCM detected subclinical extensions in only 3 out of 10 cases when compared with dermoscopically assessed margins prior to excision (30) and another demonstrated a sensitivity of 37.5% and a specificity of 95.2% for primary lateral margin detection by RCM (31). Similarly, OCT identified subclinical extensions in 11 out of 52 cases when compared with clinical and dermoscopic evaluations by a Mohs surgeon (32).

The potential advantages of HI in preoperative lateral surgical margin assessment are the field of view and the rapid image acquisition. HI can image an area up to 12 cm² in 5–10 s, while the field of view in RCM is 1 cm² (with mosaicking) in 2 min (33). OCT images areas of 6 x 6 mm (0.36 cm^2^), of which 5 x 5 mm is captured and processed in 40 s (34). The imaging depth of HI is approximately 2 mm, compared with 200 µm for RCM (10), 0.2–2.5 mm for OCT (34), and 500 µm for LC-OCT (35). The HI system’s lateral resolution is 20 μm, which is lower compared with OCT (1–15 μm), LC-OCT (1.3 μm), and RCM (1 μm). LC-OCT provides 3 imaging modes with real-time production of vertical and horizontal sectional images at 8 frames per second (35). Other non-invasive technologies include high-frequency and ultra-high-frequency ultrasound that allow for scanning depths ranging from 3.8 to 15.0 mm (36). Thus, these techniques may be more suitable for measuring tumour depth (11).

In this study’s cohort, the lateral margins were often incorrectly detected with dermoscopy with 63% (19/30) of the included cases showing subclinical lateral tumour extension. The dermoscopic evaluation was done by a Mohs surgeon with more than 10 years of experience in dermoscopy, although an evaluation by 2 experts would decrease the possibility of incorrect assessment of the dermoscopic margins.

Studies examining incomplete excision of BCCs have shown that lateral margins are affected in 52–82% of cases, deep margins are affected in 14–36%, and both margins are affected in 2–16% (3, 37–42). Nevertheless, the majority of previous studies examining which margin was affected included all subtypes without specifying the rates for each subtype. Thus, it seems especially relevant to continue developing non-invasive imaging techniques for more accurate detection of the lateral tumour extension of high-risk BCCs.

This study shows that HI is feasible for detecting the lateral borders of BCCs, with relatively high sensitivity, specificity, and overall accuracy. Previously, in addition to the margin assessment (19, 22), HI has shown potential in the evaluation of field-cancerized skin (20) and non-invasive differential diagnostic of melanocytic lesions (21, 25). HI technology is comparatively low-cost, user-friendly, and could potentially be integrated into smartphones, making it accessible for widespread use by both dermatologists and general practitioners. Further advantages of HI include automated fast non-user-dependent AI-aided analysis (not user-dependent) as well as a fast imaging process.

### Limitations

A limitation of the study is that the sample size was small, and halves of the images were used for the CNN training phase. Ideally, the CNN training should be conducted with a dataset separate from that used for the classification task. However, the pixel-wise analysis overcame this potential bias and increased the reliability of the findings. In addition to the half-split approach, we also performed leave-one-out cross-validation at the image level, where each lesion was excluded in turn. This provided a conservative estimate of generalization to unseen lesions, whereas the half-split results represent an optimistic upper bound of performance if larger datasets covering the full variability of lesions were available. Furthermore, the methodology did not permit assessment of the deep margins. Lastly, lesions located on very irregular surfaces (e.g., ears or eyelids) were excluded in our study due to technical difficulties in image acquisition. Similar challenges regarding locations to image uneven surfaces of the face have previously posed a problem with HI and have led to exclusions (43). There is a need for further development of the device for uneven surfaces, where it can also be difficult to dermoscopically delineate the tumours. One study focusing on complex surfaces with the new SICSURFIS imager are showing promising results, but further studies are needed (26).

### Conclusion

In summary, HI may reduce the number of MMS stages by preoperatively defining the lateral margins of ill-defined facial BCCs more accurately than the clinical and dermoscopic evaluation. HI might also be useful in reducing the number of re-excisions in traditional surgery by more accurate preoperative assessment if MMS is not available.

## References

[1] Lomas A, Leonardi-Bee J, Bath-Hextall F. A systematic review of worldwide incidence of nonmelanoma skin cancer. Br J Dermatol 2012; 166: 1069–1080. 10.1111/j.1365-2133.2012.10830.x 22251204

[2] Kappelin J, Nielsen K, Nilsson F, Bjellerup M, Ahnlide I. Surgical treatment of basal cell carcinoma: a case series on factors influencing the risk of an incomplete primary excision. J Eur Acad Dermatol Venereol 2020; 34: 2518–2525. 10.1111/jdv.16327 32124503

[3] Ceder H, Ekström A, Hadzic L, Paoli J. Clinicopathological factors associated with incomplete excision of high-risk basal cell carcinoma. Acta Derm Venereol 2021; 101: adv00496. 10.2340/00015555-3856 34184066 PMC9413780

[4] Peris K, Fargnoli MC, Kaufmann R, Arenberger P, Bastholt L, Seguin NB, et al. European consensus-based interdisciplinary guideline for diagnosis and treatment of basal cell carcinoma – update 2023. Eur J Cancer 2023; 192: 113254. 10.1016/j.ejca.2023.113254 37604067

[5] Kunskapbanken. Available from: https://kunskapsbanken.cancercentrum.se/diagnoser/basalcellscancer/vardprogram/.

[6] Kim JYS, Kozlow JH, Mittal B, Moyer J, Olencki T, Rodgers P. Guidelines of care for the management of basal cell carcinoma. J Am Acad Dermatol 2018; 78: 540–559. 10.1016/j.jaad.2017.10.006 29331385

[7] Reiter O, Mimouni I, Dusza S, Halpern AC, Leshem YA, Marghoob AA. Dermoscopic features of basal cell carcinoma and its subtypes: a systematic review. J Am Acad Dermatol 2021; 85: 653–664. 10.1016/j.jaad.2019.11.008 31706938 PMC9366765

[8] Ceder H, Backman E, Marghoob A, Navarrete-Dechent C, Polesie S, Reiter O, et al. Importance of both clinical and dermoscopic findings in predicting high-risk histopathological subtype in facial basal cell carcinomas. Dermatol Pract Concept 2024; 14: e2024212. 10.5826/dpc.1403a212 38934710 PMC11314728

[9] Reiter O, Mimouni I, Gdalevich M, Marghoob AA, Levi A, Hodak E, et al. The diagnostic accuracy of dermoscopy for basal cell carcinoma: a systematic review and meta-analysis. J Am Acad Dermatol 2019; 80: 1380–1388. 10.1016/j.jaad.2018.12.026 30582991

[10] Que SKT. Research techniques made simple: noninvasive imaging technologies for the delineation of basal cell carcinomas. J Invest Dermatol 2016; 136: e33–e38. 10.1016/j.jid.2016.02.012 27012561

[11] Janowska A, Oranges T, Granieri G, Romanelli M, Fidanzi C, Iannone M, et al. Non-invasive imaging techniques in presurgical margin assessment of basal cell carcinoma: current evidence. Skin Res Technol 2023; 29: e13271. 10.1111/srt.13271 36823508 PMC10155792

[12] Soglia S, Pérez-Anker J, Lobos Guede N, Giavedoni P, Puig S, Malvehy J. Diagnostics using non-invasive technologies in dermatological oncology. Cancers (Basel) 2022; 14: 5886. 10.3390/cancers14235886 36497368 PMC9738560

[13] Niculet E, Craescu M, Rebegea L, Bobeica C, Nastase F, Lupasteanu G, et al. Basal cell carcinoma: comprehensive clinical and histopathological aspects, novel imaging tools and therapeutic approaches (Review). Exp Ther Med 2022; 23: 60. 10.3892/etm.2021.10982 34917186 PMC8630439

[14] Malvehy J, Pellacani G. Dermoscopy, confocal microscopy and other non-invasive tools for the diagnosis of non-melanoma skin cancers and other skin conditions. Acta Derm Venereol 2017; Suppl 218: 22–30. 10.2340/00015555-2720 28676883

[15] De Carvalho N, Schuh S, Kindermann N, Kästle R, Holmes J, Welzel J. Optical coherence tomography for margin definition of basal cell carcinoma before micrographic surgery: recommendations regarding the marking and scanning technique. Skin Res Technol 2018; 24: 145–151. 10.1111/srt.12407 29057513

[16] Paradisi A, Cornacchia L, Cappilli S, Abeni D, Federico F, Di Stefani A, et al. Preoperative evaluation of high-risk basal cell carcinoma with line-field confocal optical coherence tomography (LC-OCT) reduces Mohs micrographic surgery stage number: a case-control study. EJC Skin Cancer 2024; 2: 100015. 10.1016/j.ejcskn.2023.100015

[17] Lu G, Fei B. Medical hyperspectral imaging: a review. J Biomed Opt 2014; 19: 10901. 10.1117/1.JBO.19.1.010901 24441941 PMC3895860

[18] Neittaanmäki N, Salmivuori M, Pölönen I, Jeskanen L, Ranki A, Saksela O, et al. Hyperspectral imaging in detecting dermal invasion in lentigo maligna melanoma. Br J Dermatol 2017; 177: 1742–1744. 10.1111/bjd.15267 28012166

[19] Neittaanmäki-Perttu N, Grönroos M, Jeskanen L, Pölönen I, Ranki A, Saksela O, et al. Delineating margins of lentigo maligna using a hyperspectral imaging system. Acta Derm Venereol 2015; 95: 549–552. 10.2340/00015555-2010 25394551

[20] Neittaanmäki-Perttu N, Grönroos M, Tani T, Pölönen I, Ranki A, Saksela O, et al. Detecting field cancerization using a hyperspectral imaging system. Lasers Surg Med 2013; 45: 410–417. 10.1002/lsm.22160 24037822

[21] Räsänen J, Salmivuori M, Pölönen I, Grönroos M, Neittaanmäki N. Hyperspectral imaging reveals spectral differences and can distinguish malignant melanoma from pigmented basal cell carcinomas: a pilot study. Acta Derm Venereol 2021; 101: adv00405. 10.2340/00015555-3755 33521835 PMC9366698

[22] Salmivuori M, Neittaanmäki N, Pölönen I, Jeskanen L, Snellman E, Grönroos M. Hyperspectral imaging system in the delineation of ill-defined basal cell carcinomas: a pilot study. J Eur Acad Dermatol Venereol 2019; 33: 71–78. 10.1111/jdv.15102 29846972

[23] Saari H, Pölönen I, Salo H, Honkavaara E, Hakala T, Holmlund C, et al. Miniaturized hyperspectral imager calibration and UAV flight campaigns. In: Sensors, systems, and next-generation satellites XVII. Proc SPIE 2013: p. 448–459. 10.1117/12.2028972

[24] Barun VV, Ivanov AP, Volotovskaya AV, Ulashchik VS. Absorption spectra and light penetration depth of normal and pathologically altered human skin. J Appl Spectroscopy 2007; 74: 430–439. 10.1007/s10812-007-0071-2

[25] Paoli J, Pölönen I, Salmivuori M, Räsänen J, Zaar O, Polesie S, et al. Hyperspectral imaging for non-invasive diagnostics of melanocytic lesions. Acta Derm Venereol 2022; 102: adv00815. 10.2340/actadv.v102.2045 36281811 PMC9811300

[26] Raita-Hakola AM, Annala L, Lindholm V, Trops R, Näsilä A, Saari H, et al. FPI based hyperspectral imager for the complex surfaces: calibration, illumination and applications. Sensors (Basel) 2022; 22: 3420. 10.3390/s22093420 35591109 PMC9103796

[27] Tan M, Le Q. EfficientNet: rethinking model scaling for convolutional neural networks. In: Kamalika C, Ruslan S, editors. Proceedings of the 36th International Conference on Machine Learning, 2019: p. 6105–6114.

[28] Yeom SD, Lee SH, Ko HS, Chung KY, Shin J, Choi GS, et al. Effectiveness of dermoscopy in Mohs micrographic surgery (MMS) for nonmelanoma skin cancer (NMSC). Int J Dermatol 2017; 56: e136–e139. 10.1111/ijd.13501 28247925

[29] Adan F, Kallen EJJ, Dermont G, Muche JM, Sinx KAE, Schilder A, et al. Diagnostic accuracy of optical coherence tomography in the assessment of in vivo primary basal cell carcinoma resection margins prior to Mohs Micrographic Surgery. J Eur Acad Dermatol Venereol 2022; 36: e270–e272. 10.1111/jdv.17804 34784080

[30] Venturini M, Gualdi G, Zanca A, Lorenzi L, Pellacani G, Calzavara-Pinton PG. A new approach for presurgical margin assessment by reflectance confocal microscopy of basal cell carcinoma. Br J Dermatol 2016; 174: 380–385. 10.1111/bjd.14244 26498991

[31] Lupu M, Voiculescu VM, Caruntu A, Tebeica T, Caruntu C. Preoperative evaluation through dermoscopy and reflectance confocal microscopy of the lateral excision margins for primary basal cell carcinoma. Diagnostics (Basel) 2021; 11: 120. 10.3390/diagnostics11010120 33466602 PMC7828674

[32] Wang KX, Meekings A, Fluhr JW, McKenzie G, Lee DA, Fisher J, et al. Optical coherence tomography-based optimization of Mohs micrographic surgery of basal cell carcinoma: a pilot study. Dermatol Surg 2013; 39: 627–633. 10.1111/dsu.12093 23293854

[33] Larson B, Abeytunge S, Seltzer E, Rajadhyaksha M, Nehal K. Detection of skin cancer margins in Mohs excisions with high-speed strip mosaicing confocal microscopy: a feasibility study. Br J Dermatol 2013; 169: 922–926. 10.1111/bjd.12444 23701464 PMC3867275

[34] Cheng HM, Guitera P. Systematic review of optical coherence tomography usage in the diagnosis and management of basal cell carcinoma. Br J Dermatol 2015; 173: 1371–1380. 10.1111/bjd.14042 26211438

[35] Ruini C, Schuh S, Sattler E, Welzel J. Line-field confocal optical coherence tomography: practical applications in dermatology and comparison with established imaging methods. Skin Res Technol 2021; 27: 340–352. 10.1111/srt.12949 33085784

[36] Levy J, Barrett DL, Harris N, Jeong JJ, Yang X, Chen SC. High-frequency ultrasound in clinical dermatology: a review. Ultrasound J 2021; 13: 24. 10.1186/s13089-021-00222-w 33877462 PMC8058126

[37] Bassas P, Hilari H, Bodet D, Serra M, Kennedy FE, García-Patos V. Evaluation of surgical margins in basal cell carcinoma by surgical specialty. Actas dermo-sifiliograficas 2013; 104: 133–140. 10.1016/j.ad.2012.06.001 22835227

[38] Griffiths RW. Audit of histologically incompletely excised basal cell carcinomas: recommendations for management by re-excision. Br J Plast Surg 1999; 52: 24–28. 10.1054/bjps.1998.3018 10343586

[39] Kumar P, Orton CI, McWilliam LJ, Watson S. Incidence of incomplete excision in surgically treated basal cell carcinoma: a retrospective clinical audit. Br J Plast Surg 2000; 53: 563–566. 10.1054/bjps.2000.3394 11000071

[40] Masud D, Moustaki M, Staruch R, Dheansa B. Basal cell carcinomata: risk factors for incomplete excision and results of re-excision. J Plast Reconstr Aesthet Surg 2016; 69: 652–656. 10.1016/j.bjps.2015.12.024 26948998

[41] Dieu T, Macleod AM. Incomplete excision of basal cell carcinomas: a retrospective audit. ANZ J Surg 2002; 72: 219–221. 10.1046/j.1445-2197.2002.02351.x 12071456

[42] Su SY, Giorlando F, Ek EW, Dieu T. Incomplete excision of basal cell carcinoma: a prospective trial. Plast Reconstr Surg 2007; 120: 1240–1248. 10.1097/01.prs.0000279148.67766.e1 17898596

[43] Christensen GB, Nagaoka T, Kiyohara Y, Johansson I, Ingvar C, Nakamura A, et al. Clinical performance of a novel hyperspectral imaging device for cutaneous melanoma and pigmented skin lesions in Caucasian skin. Skin Res Technol 2021; 27: 803–809. 10.1111/srt.13023 33651425

